# When is a autologous bone marrow transplantation indicated in the treatment of juvenile systemic sclerorsis? Results of a multinational survey of Pediatric Rheumatologist

**DOI:** 10.1186/1546-0096-9-S1-P73

**Published:** 2011-09-14

**Authors:** Ivan Foeldvari, Angela Wierk, Dominique Farge

**Affiliations:** 1 Hamburger Zentrum für Kinder- und Jugendrheumatologie, Hamburg , Germany; 2 Rheumatology, Hopital Saint Louis, Paris, France

## 

The five year survival of juvenile systemic sclerosis (jSSc) is around 95%. Patients, who died in the two retrospective cohorts, died mostly in the first 24 months of disease course. Autologous bone marrow transplantation (ABMT) seems to be a promising therapeutic approach for adult patients ( ASTIS (EU) and SCOT (USA)Trial) with severe disease course. Around 8 patients with jSSc are transplanted according the EBMT registry. Currently no consent based inclusion or exclusion criteria for ABMT in jSSc exists.

Aim of the survey was to get a feeling from paediatric rheumatologists, when they would apply autologous bone marrow transplantation as a treatment option.

Paediatric Rheumatologist - members of the PRES Juvenile Scleroderma Working Group and participants of the Paediatric Rheumatology E-mail Board were asked via Internet to fill out the survey

22 centres responded, all of them were academic centres. BMT would be considered for 14 of the 22 colleagues after nonresponse to cyclophosphamide, 10 of 22 after non response to two DMARDs and 12 of 22 after nonresponse to Rituximab. 19 of 22 would consider transplantation if the CHAQ score ≤ 2 , 20 of 22 if the CHQ is less than 40%. 21 of 22 would think about transplantation if the modified Rodnan skin score is more than 30 and 15 of 22 if the DLCO is less than 50%, 18 of 22 if the WHO functional class is 3, 14 of 22 if the FVC less than 60%, 15 of 22 if the pulmonary arterial pressure more than 40 mm/hg and 11 of 22 if left ventricular ejection fraction is less than 40%.

This survey represents an impression, when pediatric rheumatologist would consider ABMT. It is a starting point for a possible evolving ABMT program for this orphan disease

